# A Web-Based Tool for Patient Triage in Emergency Department Settings: Validation Using the Emergency Severity Index

**DOI:** 10.2196/medinform.3508

**Published:** 2015-06-10

**Authors:** Pierre Elias, Ash Damle, Michael Casale, Kim Branson, Chaitanya Churi, Ravi Komatireddy, Jamison Feramisco

**Affiliations:** ^1^ Duke Clinical Research Institute Duke University School of Medicine Durham, NC United States; ^2^ Lumiata, Inc San Mateo, CA United States; ^3^ Clinical Research Division West Health Institute La Jolla, CA United States; ^4^ University of Texas Health Science Center Houston, TX United States; ^5^ Clinical Research Division Scripps Translation Science Institute La Jolla, CA United States; ^6^ Lumiata, Inc. San Mateo, CA United States

**Keywords:** triage, emergency severity index, differential diagnosis, clinical decision support

## Abstract

**Background:**

We evaluated the concordance between triage scores generated by a novel Internet clinical decision support tool, Clinical GPS (cGPS) (Lumiata Inc, San Mateo, CA), and the Emergency Severity Index (ESI), a well-established and clinically validated patient severity scale in use today. Although the ESI and cGPS use different underlying algorithms to calculate patient severity, both utilize a five-point integer scale with level 1 representing the highest severity.

**Objective:**

The objective of this study was to compare cGPS results with an established gold standard in emergency triage.

**Methods:**

We conducted a blinded trial comparing triage scores from the ESI: A Triage Tool for Emergency Department Care, Version 4, Implementation Handbook to those generated by cGPS from the text of 73 sample case vignettes. A weighted, quadratic kappa statistic was used to assess agreement between cGPS derived severity scores and those published in the ESI handbook for all 73 cases. Weighted kappa concordance was defined a priori as almost perfect (kappa > 0.8), substantial (0.6 < kappa < 0.8), moderate (0.4 < kappa < 0.6), fair (0.2 < kappa< 0.4), or slight (kappa < 0.2).

**Results:**

Of the 73 case vignettes, the cGPS severity score matched the ESI handbook score in 95% of cases (69/73 cases), in addition, the weighted, quadratic kappa statistic showed almost perfect agreement (kappa = 0.93, 95% CI 0.854-0.996). In the subanalysis of 41 case vignettes assigned ESI scores of level 1 or 2, the cGPS and ESI severity scores matched in 95% of cases (39/41 cases).

**Conclusions:**

These results indicate that the cGPS is a reliable indicator of triage severity, based on its comparison to a standardized index, the ESI. Future studies are needed to determine whether the cGPS can accurately assess the triage of patients in real clinical environments.

## Introduction

### Emergency Department Medical Triage for Patients

Accurate medical triage is critical to patient management in environments such as urgent care centers and emergency departments.  Previous research has shown that matching the supply and demand of medical resources within the emergency department (ED) is a complex task with many competing variables [1,2]. Errors in the initial clinical evaluation of patients can potentially lead to severe consequences such as a misdiagnosis, delayed treatment, disproportionate health care resource utilization, and increased costs [3,4].  Over the past three decades, two particular developments have significantly contributed to improved triage.

### The Use of Standardized Triage Protocols

The first such development toward improved ED triage was the introduction of standardized protocols such as the Ipswich triage scale, the Australasian triage scale, the Canadian Association of Emergency Physicians triage scale, and the Emergency Severity Index (ESI), which have provided triage templates aimed at consistency and reproducibility across a wide array of patient presentations [5-8]. The ESI, a 5-level triage scoring system, provides a standardized and experimentally validated method of assigning risk severity to patients based upon assessment of their complaints, relevant history, and vital signs when appropriate. Additionally, the anticipated resource utilization is considered [9]. The ESI 5-level triage methodology has been validated when considering resource utilization in diagnostic testing, consultation, and admission to an inpatient setting, as well as 6-month post clinical-evaluation morbidity and mortality [10]. For this evaluation, the ESI was treated as the reference standard for assigning appropriate triage.

### Electronic Tools and Patient Data

The second development was the creation and adoption of electronic tools in the form of electronic health records, utilization review software, and clinical decision support (CDS) systems. These tools have changed the way medical professionals manage patient data, communicate with patients and other health care providers, and consider diagnostic and therapeutic options. Preliminary research on CDS technology suggests that this type of medical guidance may play a significant role in patient management and triage, particularly in clinical areas such as EDs, which are characterized by high patient volume, time pressure, and varied pathologies and severities [11,12].

There is significant potential to improve the triaging process through automation. Triaging represents a costly bottleneck in hospital throughput; a 2009 study of Pennsylvania ED directors found that 83% agreed that ED overcrowding was a problem in their hospitals [13]. Such challenges have worsened in recent years; from 1995 to 2005, annual ED visits in the United States increased by 20% (from 96.5 to 115.3 million), and the ED utilization rate increased by 7% (from 36.9 to 39.6 ED visits per 100 persons) [14]. Despite increasing ED visits, the number of hospital EDs decreased by 381, and total hospital beds decreased by 134,000 during the same decade [14].

In part by automating time-intensive tasks, computerized CDS tools for triage aim to improve expediency, patient outcomes, and hospital throughput. Numerous systems have attempted to develop computerized CDS for triage with some success [11,15]. The Taiwan Triage and Acuity Scale was able to significantly decrease overtriaging and medical resource consumption in one study of its implementation [16]. Yet, despite significant advancements, current computerized CDS triage tools suffer significant limitations. First, they are only able to incorporate structured data. This represents a significant workflow restriction; health care provider notes, which provide some of the most valuable information for triaging, are not utilized. Second, agreement of these tools compared to chart review is often poor to moderate. A systematic review found kappa ranges from 0.2 to 0.87, with most below 0.5 [15].

In this study, we evaluated concordance between an established triage severity scoring system, the ESI, and a 5-level triage score created by a novel CDS tool, Clinical GPS v2.0 from Lumiata Inc (cGPS).  Although cGPS is designed to be used with electronic health records utilizing an application programming interface (API), it is not yet available to the public, since it is still in the development stage and it has not yet been evaluated by the US Food and Drug Administration. Physician providers are currently testing it in multiple health care settings; the publication of the results of those validation studies is planned.

Using a proprietary database of physician-curated medical information, the cGPS produces a triage severity score based upon a patient’s demographics, clinical objective signs, subjective symptoms, vital signs, objective laboratory data, past medical history, and medications (note that laboratory data, past medical history, and medications are not required inputs in the triage setting). The cGPS tool aggregates these data, performs its analysis, and then constructs a list of probable diagnoses from a graph-structure database, each of which has an associated triage score range. All clinical inputs and differential diagnosis lists are utilized to arrive at a single whole-number triage score. In this study, we sought to pursue an independent evaluation against a reference standard in triage (ESI) to validate the potential for future clinical use of cGPS in actual health care settings. This blinded study compared cGPS-generated triage scores for 73 sample case vignettes from the Emergency Severity Index, Version 4: Implementation Handbook (Agency for Healthcare Research and Quality, 2005) [17] against the “gold-standard” ESI-created scores found in the Handbook.   

## Methods

### Methodology of the Clinical GPS and Emergency Severity Index Algorithms

The cGPS database was created through physician curation, which attempts to connect the data inputs physicians receive directly from patients with all the knowledge, data, and experience health professionals have acquired over the years. Multiple physicians began with a list of signs and symptoms associated with a given diagnosis. Each physician would then remove and add symptoms based on their experience, available data, and published guidelines. This process was followed by adding associated ranges of severity and frequency (eg, severe cough is a common symptom of the diagnosis asthma).

The cGPS system then uses multi-dimensional probability distribution to build graph representations of how illnesses and patients are connected. The cGPS algorithm is based on a probabilistic graphical model, or graph analysis. Graph analysis is a technique for making sense of large datasets, primarily by determining how similar data points are among a range of parameters. The graph’s nodes include diagnoses, objective signs, symptoms, laboratory test results, vital signs, and other common inputs to medical decision making. The edges between nodes are probabilistic and based on demographic factors, including age, gender, race, and duration of symptoms. For example, the baseline probability of “abdominal pain” being a presentation of the diagnosis “appendicitis” increases or decreases depending on factors such as the patient’s age, gender, and the duration of the symptom.

Each sign and symptom in the cGPS system was assigned a range of potential severity scores from 1-5 by a physician using the working definition highlighted in Table 1. Physicians also manually curated the relationships between diagnoses, signs, and symptoms, and assigned a frequency category, as seen in Table 2. The physician-curated frequency and severity categories are then combined to generate probability distribution scores per diagnosis. For example, “tearing chest pain” may be considered critical and assigned a score of 2, but because it is a rare symptom for appendicitis, it would not heavily contribute to the overall cGPS score of the diagnosis. Another example is the diagnosis of “pneumonia” presenting with the symptom “confusion”. This combination is significantly more common in younger patients and older patients, and less prevalent for ages in between. While the severity of confusion as a symptom of pneumonia would be input as an equally severe score of 3 across all ages, it would be input as more common for the young and elderly.

The ESI algorithm categorizes ED patients by first assessing acuity level, followed by expected resource needs. Acuity is determined by the stability of vital functions and the potential threat to life, limb, or organ. Expected resource needs are defined as the number of resources a patient is expected to consume in order to arrive at a disposition decision (discharge, admission, or transfer). Triage personnel work through an algorithm of four decision points to arrive at the ESI severity score and triage level [17].

**Figure 1:**
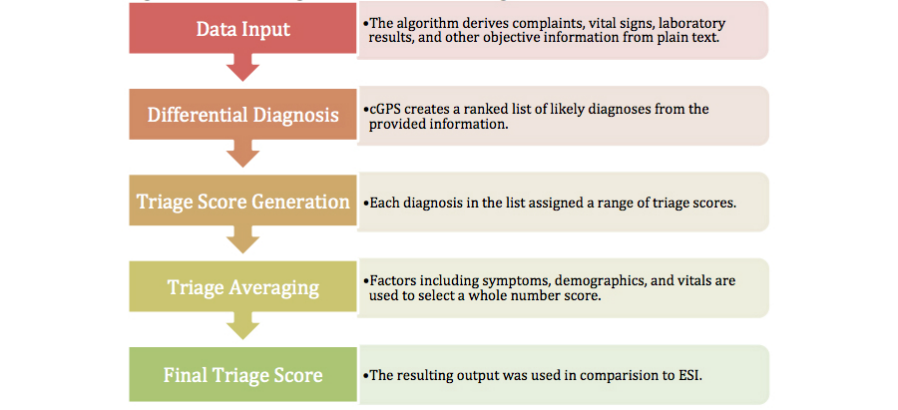
Overview of the algorithm used to derive the triage score.

**Figure 2:**
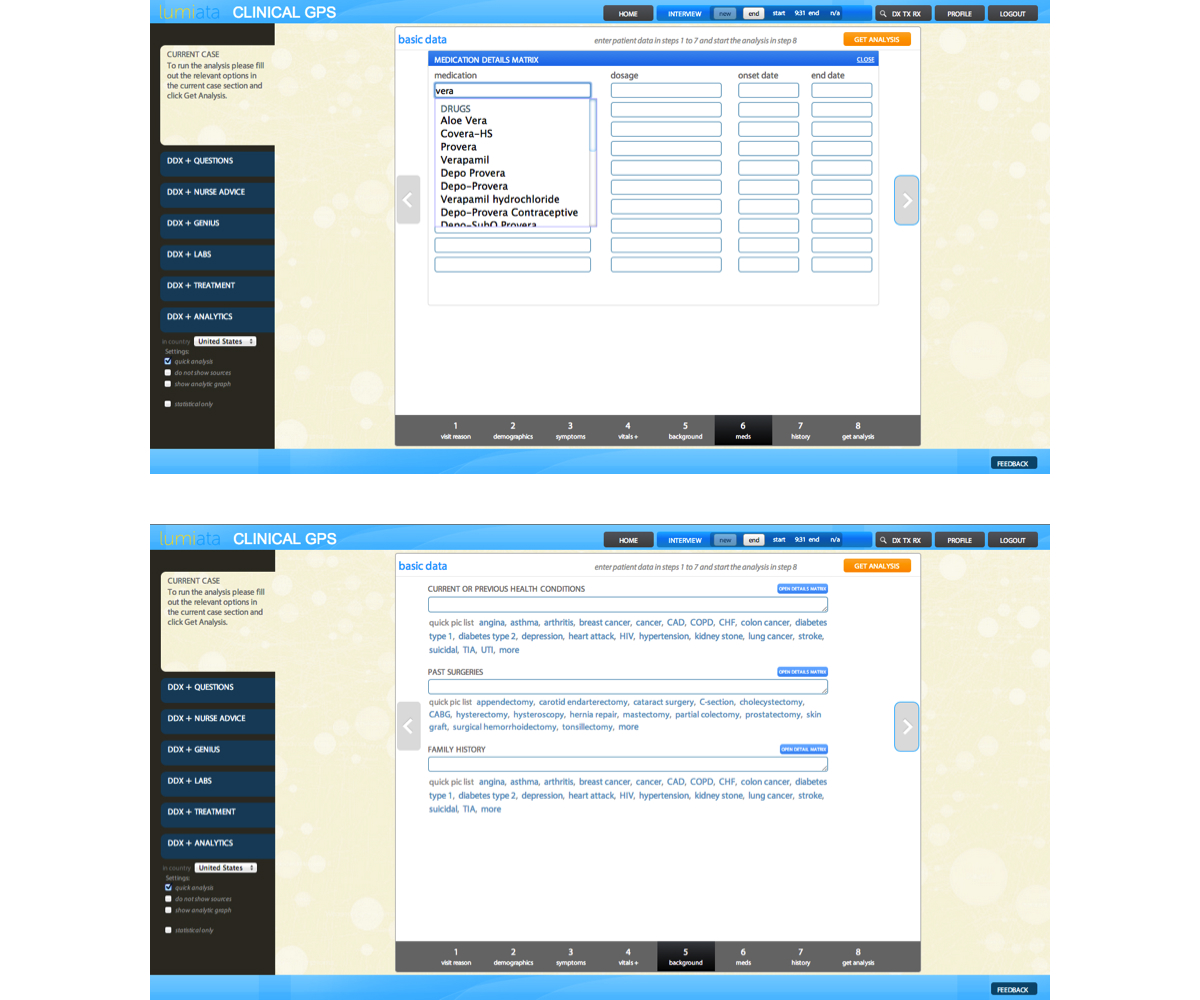
The clinical GPS v2.0 (cGPS) Web-based tool takes clinicians through an 8-step process that supports natural language entry (A) and uses autosuggestions and “quick picks” to maximize efficiency (B).

Both the ESI and cGPS utilize a 5-point scale, with 1 representing the highest severity level and 5 representing the lowest. The definitions of each triage score are similar between the ESI and cGPS, and thus were assumed to be roughly equivalent. The 5-point ESI and cGPS scales are detailed in Table 1. The descriptions for the cGPS severity scores were chosen during the initial development of the program. A key difference between the scales is that cGPS allows fractional scores (eg, 4.3) in the preliminary stage. All such fractions were converted to integer values before comparing the scores (see the Study Methodology subsection below; Figure 1 shows the algorithm below and Figure 2 shows the cGPS interface.).

**Table 1:** Working definitions used to describe each level of severity.

Severity score	ESI (text descriptors are extrapolated from Figure 2-1A) [17]	cGPS
1	Immediate lifesaving intervention required	Revive/unstable
2	High-risk situation or confused/lethargic/disoriented or severe pain/distress	Critical
3	Urgent, complex (2 or more resources)	Urgent
4	Nonurgent, less complex (1 resource)	Nonurgent
5	Nonurgent (no resources)	Referred

### Clinical GPS and Emergency Severity Index Algorithm Differences

**Figure 3:**
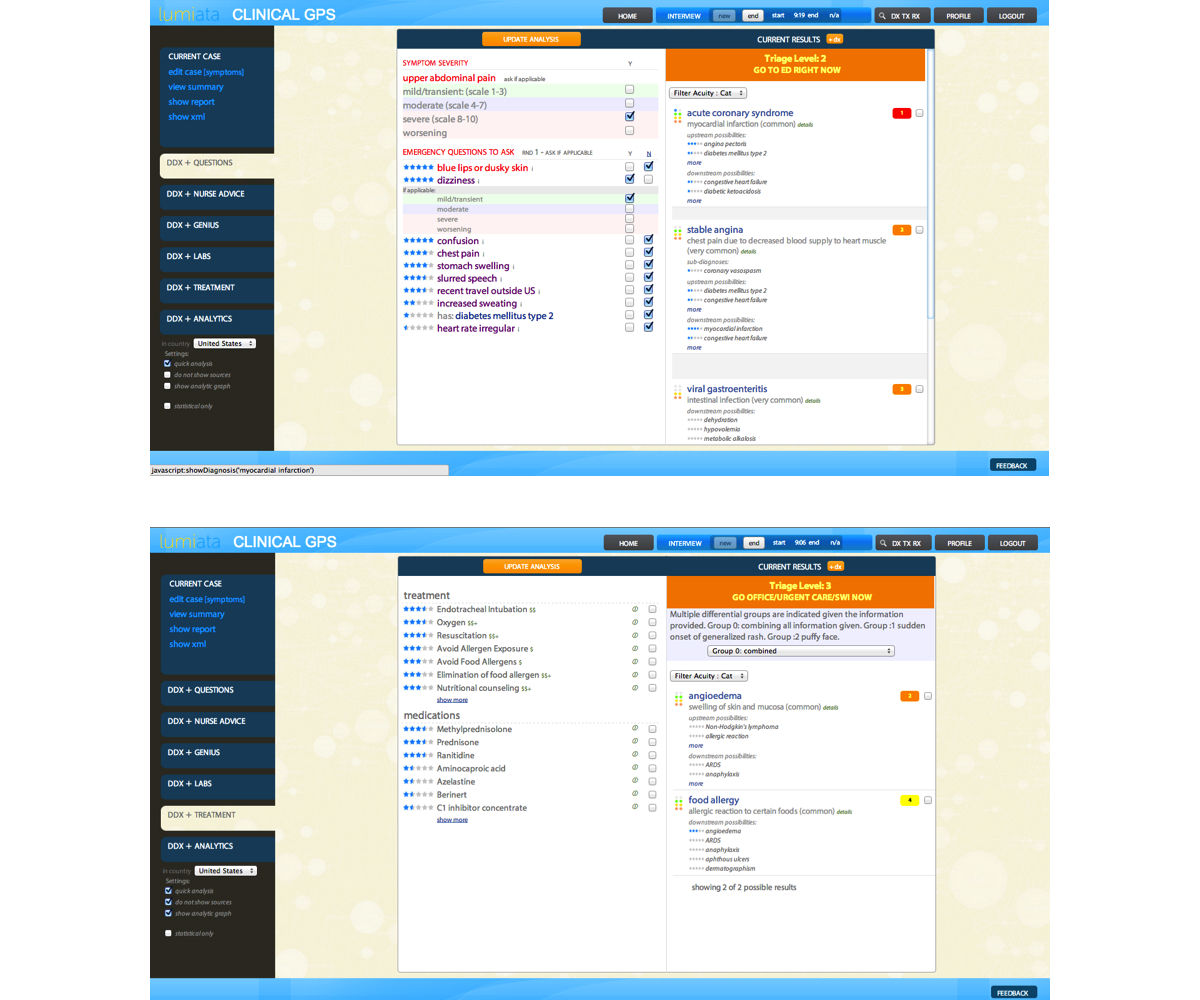
The clinical GPS v2.0 (cGPS) generates differential diagnoses with severity scores and upstream and downstream possibilities, and follow-up questions and tests, including associated costs (C & D).

As detailed above, the methods used by the ESI and cGPS to arrive at these scores are fundamentally different. The ESI score utilizes acuity information in addition to projected resource utilization to arrive at a triage score. In contrast, for each set of signs and symptoms presented in the input, the cGPS produces a list of differential diagnoses (Figure 3 shows this) using the algorithm detailed below (shown in Figure 1). Because the approaches are fundamentally untethered, no ESI data were used for training the cGPS algorithm prior to the study.

**Table 2:** cGPS’s physician-curated signs and symptoms frequency categories.

Frequency category	Description
Key	Required for diagnosis
Very common	Occurs in >50% of presentations for diagnosis
Common	Occurs in 10-50% of presentations for diagnosis
Uncommon	Occurs in 1-10% of presentations for diagnosis
Rare	Occurs in <1% of presentations for diagnosis

                                                                                                       

### Study Methodology

For this study, 73 sample patient case vignettes from the ESI handbook were entered verbatim into the cGPS.  No case vignettes were excluded from entry. Next, the corresponding ESI score for each vignette was taken directly from the ESI handbook, and the blinded scores were compared. As no human subject or actual patient medical information was used in this analysis, institutional review board approval and patient consent were not applicable.

**Figure 4:**
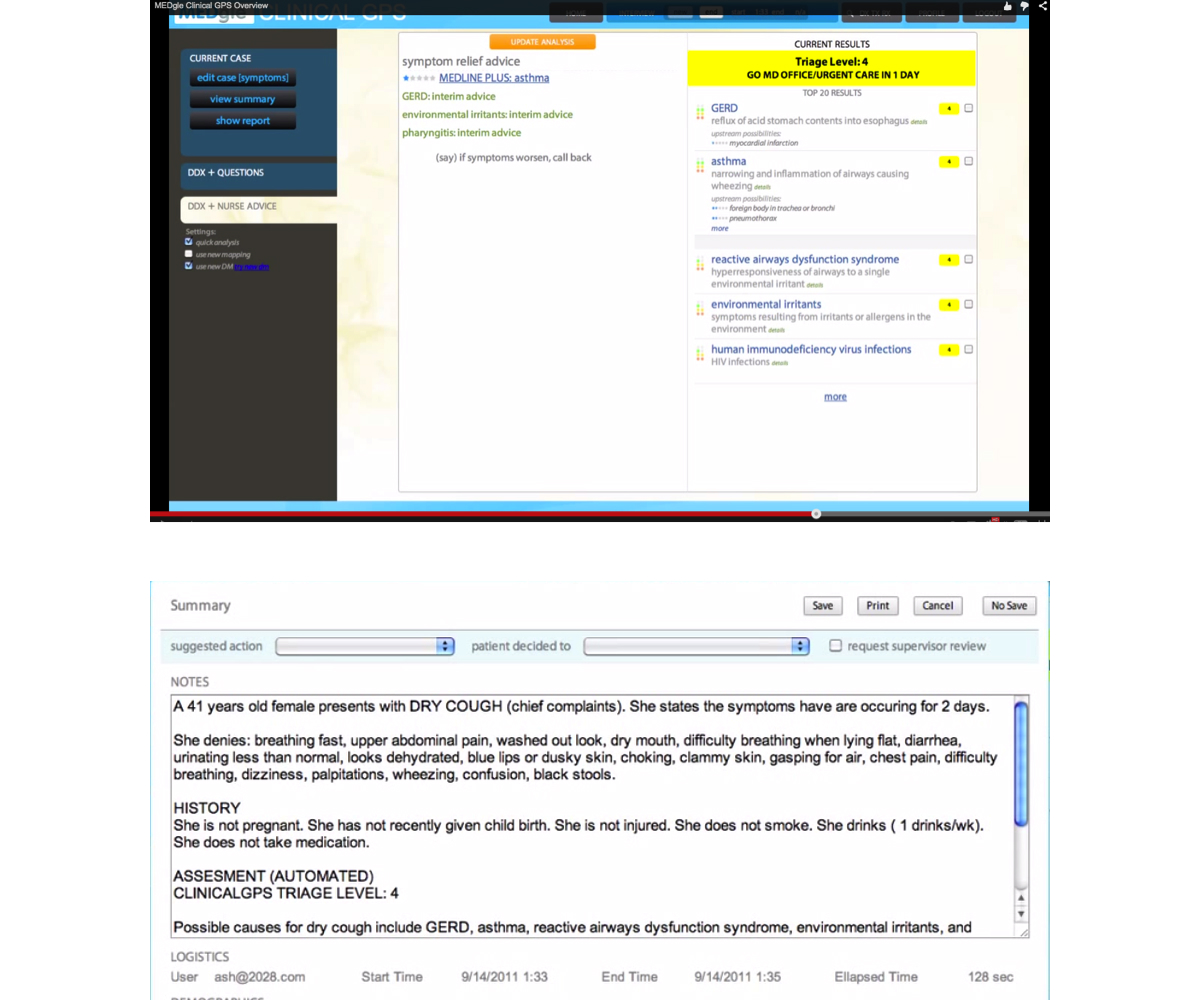
The clinical GPS v2.0 (cGPS) interfaces directly with the electronic health record (E & F).

For each case vignette, the ESI handbook provides a severity score that was obtained using the ESI triage methodology. Vignette topics cover a wide array of potential pathologies and severities. The ESI handbook does not list specific differential diagnoses for each vignette, nor do the vignettes present objective laboratory data; however, all vignettes include the patient’s age, gender, and at least one sign or symptom. Whenever present, patient’s complaints, past medical history, vital signs, and physical exam values were entered into the cGPS program exactly as written in the handbook. The narrative nature of the handbook vignettes was compatible with the cGPS user interface that allows users to input core information in a format similar to the history and physical note that physicians commonly use to document care (Figures 2 and 4 show the interface). Thus, for each ESI vignette, the data available in the handbook were entered directly into the cGPS tool, and the cGPS algorithm then generated a triage score.

This preliminary severity score generated by the cGPS algorithm was a value ranging from 1 through 5 that included a fractional component and was converted to a whole number value. Average scores with a fraction that fell within the middle range of 0.40-0.60, for example 3.5, required an extra step for rounding. For these middle range scores in the cGPS, the list of recommended diagnostic tests and the duration of symptoms were examined.  

The presence of one or more testing modalities considered to be critical with regard to a specific urgent/emergent diagnosis (eg, electrocardiogram for chest pain), as well as a short duration of symptoms, caused the average severity score to be rounded to the next highest triage level. Conversely, the lack of any suggestions for critical testing modalities or a long duration of symptoms resulted in the score being rounded down to the next lower triage level. Figure 1 shows an overview of the cGPS algorithm used to derive a triage score. The cGPS utilized the individual whole number severity scores for the top 100 diagnoses in the differential diagnosis list to create an average severity score. It was estimated that using 100 diagnoses would account for most physicians’ lists of differential diagnoses with a *P*<.001. The individual integer severity scores for each diagnosis were weighted by the baseline prevalence of each diagnosis combined with the likelihood it would present with the given signs, symptoms, and vitals.

Determinants of the severity score for each diagnosis as well as a list of suggested testing modalities were determined a priori using a medical database constructed using expert level knowledge and clinical experience. Data were collected in a spreadsheet and subsequently analyzed. The fraction of exact matches between both severity scores was calculated for all 73 cases. Additionally, a weighted, quadratic kappa statistic was calculated to assess agreement between the cGPS and ESI handbook for all 73 cases, as well as a subset of 41 cases determined to be severity level 1 and 2 in the handbook.

A quadratic kappa statistic was used because the end value of agreement is adjusted based upon the degree of disparity between the two scores. Triage scores that were several categories apart, for example, were considered to exhibit nonlinear disagreement. For example, a situation with an assessed severity of 3, but actual severity of 1, is a potentially life-ending mistake. As such, we chose to use quadratic kappa to account for the significant impact of errors several categories apart. Levels of agreement for the weighted, quadratic kappa analysis were defined a priori based upon previous research to facilitate comparisons; values < 0 indicated no agreement, 0-0.20 slight, 0.21-0.40 fair, 0.41-0.60 moderate, 0.61-0.80 substantial, and 0.81-1.0 almost perfect agreement [18-24]. Although the highest level of agreement possible was the overall goal, we considered a level of agreement above “moderate” (kappa > 0.6) as evidence of sufficient potential to pursue further improvement and testing of this triage algorithm.

## Results

### Clinical GPS and Emergency Severity Index Algorithm Scores

**Figure 5:**
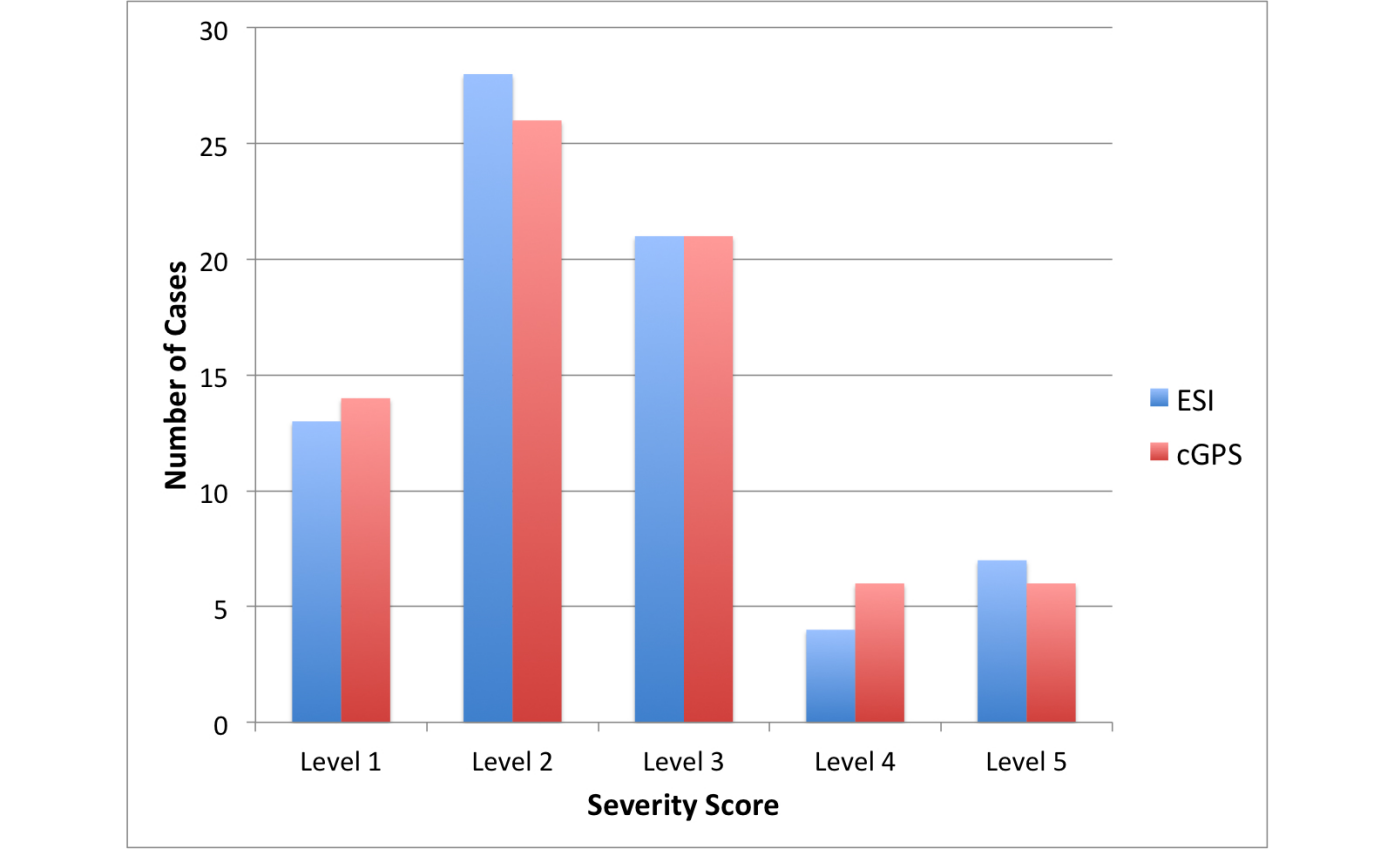
Distribution of Emergency Severity Index (ESI) and clinical GPS v2.0 (cGPS) severity scores for the case vignettes (n=73).

Of the 73 ESI handbook clinical case vignettes, the cGPS severity score perfectly matched the ESI handbook severity score in 95% of the cases (69/73 cases). The weighted, quadratic kappa statistic was kappa = 0.93 (95% CI 0.854-0.996), as Figure 5 illustrates, and as summarized in Table 3.  

**Table 3:** Results of the analysis using all case vignettes (n=73).

Results	
Matched: cGPS = ESI	69
Unmatched: cGPS ≠ ESI	4
Total number of cases	73
Percentage of cases with identical severity score	95%
Weighted, quadratic kappa	0.933 (95% CI 0.854-0.996)

### A Subanalysis of Case Vignettes

A subanalysis of 41 case vignettes that were assigned the two highest severity scores by the ESI, levels 1 and 2, showed that the cGPS and ESI scores matched exactly in 95% of cases (39/41 cases). A weighted, quadratic kappa statistic for this subgroup was 0.85 (95% CI 0.750-1.037), as shown in Table 4. However, although the quadratic kappa statistic for all 73 cases and the 41 cases in the high level subgroup were greater than 0.8, the lower limit of the CI for the subgroup falls below 0.8, rendering it not significantly better than “substantial” agreement. There were four clinical case vignettes that differed in severity scores between the two systems. In two cases, the cGPS assigned a higher severity score than that of the ESI handbook; it assigned a lower severity score than the ESI handbook in the remaining two cases (Table 5).

**Table 4:** Subgroup analysis of case vignettes determined to be severity level 1 or 2 by the ESI system (n=41).

Results	
Matched: cGPS = ESI	39
Unmatched: cGPS ≠ ESI	2
Total number of cases	41
Percentage of cases where scores were identical	0.947
Weighted, quadratic kappa	0.851 (95% CI 0.750-1.037)

**Table 5:** A matrix representation of severity score distribution between the ESI and calculated triage score.

		ESI				
		1	2	3	4	5
cGPS	1	13	1	0	0	0
	2	0	26	0	0	0
	3	0	0	20	0	1
	4	0	1	1	4	0
	5	0	0	0	0	6

## Discussion

### Principal Findings

Lumiata’s cGPS v2.0 application aims to provide patient-specific diagnostic and treatment information based upon both subjective and objective patient data.  A novel feature of this software package is its ability to infer a 5-level, ESI-equivalent triage score for patients with a broad range of clinical pathologies and severities. The cGPS exhibited a high level of agreement with the ESI when applied to the 73 sample case vignettes, despite relying upon a very different core algorithm to derive triage scores. Specifically, the cGPS generated an average triage score by analyzing an explicitly created differential diagnosis based on data input into the Internet system. Each diagnosis was assigned an individual triage score. As described in the Methods section, these scores were averaged to produce a final triage score. The cGPS produced reasonable, reliable triage scores when applied to sample cases.

In this investigation, the excellent agreement between the severity indices suggests that, in a clinical environment, the cGPS would assign a triage score similar to that assigned by the ESI methodology the majority of the time using a similar 5-point severity scale. Furthermore, there was also good agreement in triage scores for the subgroup of level 1 and 2 cases, which suggests the cGPS shows promise in identifying cases with life-threatening pathologies that require urgent or emergent medical attention. To that end, the cGPS shows significant clinical potential for evaluating triage. Further investigation is required to determine the accuracy and efficacy in a clinical setting where it could augment clinical judgment or existing triage tools such as the ESI. Importantly, the clinical consequences of assigning an incorrect triage score, even in a minority of cases, could result in significant diagnostic and treatment delays. This is especially concerning for clinical cases incorrectly assigned a lower triage severity. Similarly, an incorrect assignment of a case with low severity to a high severity category could result in inappropriate resource utilization and overall delays in the diagnosis and treatment for other patients in a queue. Although this analysis was not designed to test the accuracy of the differential diagnoses generated by cGPS, the high level of agreement between the cGPS and ESI suggests the individual triage scores assigned for each item in the differential diagnosis were accurate. It is unclear whether this suggests relevancy of the differential diagnosis itself.

We examined the four case vignettes in which the cGPS assigned a different triage score than the ESI. For the two cases where the cGPS assigned a greater severity level than the ESI handbook, one case was due to a difference in sensitivity with regard to the interpretation of vital signs, where cGPS assumed greater severity due to a minor aberrance in heart rate. The other case was due to a lower threshold of safety assumed by the cGPS in a pediatric patient.  Both of these cases were considered borderline between two discrete levels of severity, which resulted in the cGPS erring on a higher severity score for presumed safety.  Research on the validity and reliability of the ESI in pediatric cases has found that pediatric patients are more often incorrectly triaged than adult patients and, overall, patients under age 18 show the largest variation in triage decision [25]. These results highlight the importance of high-quality, reliable CDS to back up ED personnel, who may feel less comfortable making triage acuity decisions about children, especially infants. In the two cases where the GPS assigned a lower severity than the ESI handbook, the underlying reason appeared to be related to a lack of information in the cGPS database that correlates specific signs or symptoms to more severe diagnoses.  For example, one of the cases involved the eye of a construction worker being exposed to concrete. The cGPS database did not categorize concrete exposure as a chemical (alkali) splash to eye, a time-sensitive threat to life or organ, which would constitute a very-high-priority level-2 patient in both the cGPS and ESI systems. Because the cGPS database did not include concrete exposure as an alkali splash, it was unable to correctly identify it as a case requiring a higher severity score than it was assigned. Continual improvements to the underlying database of medical pathologies, as well as the incorporation of stricter rules regarding the interpretation of vital signs, would help reduce the frequency of similar inappropriate triage in the future.

ED staff deal with issues that range from overcrowding to emerging infectious diseases and natural disasters. It is important that they have access to a reliable, accurate, easy-to-use triage system. The cGPS tool meets those needs, while fitting well within the existing workflow of triage management for multiple reasons. Nurses, both in the ED, as well as over the phone, currently do most triage. The tool allows nurses to quickly and effectively document a patient’s current state within minutes, as they normally would, while receiving much more robust CDS than is currently available within most available systems. In testing, use of the cGPS tool averages 6 minutes, which is similar to other CDS systems [26] and is less than the amount of time that entering documentation into an electronic health record (EHR) typically takes [27]. Furthermore, the fact that cGPS follows standard documentation pathways, specifically the history and physical notes physicians write, makes the use and integration of such a system significantly easier. Perhaps the most important aspect for usability is the recognition that most institutions are not interested in stand-alone dashboards. If a CDS is not embedded within the EHR, it is unlikely to find interest or support. The cGPS tool is meant to be used within EHRs, utilizing an API, with future plans to communicate using Fast Healthcare Interoperability Resources to allow standardized communication with all mainstream EHRs.

### Limitations

There are several important limitations to this analysis.  The case vignettes presented in the ESI handbook were assumed to represent reasonable examples of real-life encounters with patients in a triage environment, although this assumption was not specifically validated.  A potential confounder includes data entry into the cGPS.  To maintain consistency, only one researcher was used for data entry; however, this also creates an opportunity to introduce systematic error into the data entry process. Also, there are several different methods we could have used to round the average severity scores from values with fractions to a whole number value from 1 through 5. We used what we considered to be a reasonable algorithm that would theoretically err on the side of safety and resolve ambiguity by rounding to the next higher severity. However, other methods and any associated differences in results were not explored. In addition, the generalizability of these results to an actual patient population is limited given the inherent medical complexities present in a real clinical environment, as demonstrated in previous research with electronic triage tools and simulated cases [8,9]. Finally, this study assumes that the 5-point scales used by the cGPS and the ESI are roughly equivalent with respect to identifying levels of illness and patients’ needs, although the clinical features that form the boundaries of each triage level may be subject to end-user interpretation. For the subgroup analysis of level 1 and 2 cases, we feel that increasing the sample size would aid in better characterizing the significance of the kappa statistic in this patient population.

### Conclusions

Despite a high level of agreement among the 73 cases and the subgroup with the most severe cases, the clinical consequences of incorrectly triaging even a minority of cases do not justify the use of cGPS in a clinical environment without experienced clinician guidance and comparative measures. We would expect that prior to any use in an actual clinical setting, all triage subgroups would meet at least an almost perfect level of triage score agreement with an established standard such as the ESI. In addition, the use of cGPS in any clinical setting would also require establishing its safety and reliability with a controlled clinical pilot evaluating patient cases in parallel with an established triage tool and in a prospective manner with different users and patient populations as well as clinician oversight.

This initial investigation suggests that an automated tool providing computerized CDS has potential to serve as an independent triage tool for use by providers in urgent care and emergency room settings. However, additional prospective pilot clinical studies and database improvements will be required to determine the triage accuracy with regard to real patient cases, various score algorithms, data entry procedures, performance and usability within clinical environments, and interobserver agreement between different users in clinical environments.

## Abbreviations

API: application programming interface
CDS: clinical decision support
cGPS: clinical GPS v2.0
ED: emergency department
EHR: electronic health record
ESI: Emergency Severity Index
