# Metadata Correction: A Web-Based Tool for Patient Triage in Emergency Department Settings: Validation Using the Emergency Severity Index

**DOI:** 10.2196/medinform.4816

**Published:** 2015-08-11

**Authors:** Pierre Elias, Ash Damle, Michael Cassale, Kim Branson, Nick Peterson, Chaitanya Churi, Ravi Komatireddy, Jamison Feramisco

**Affiliations:** ^1^ Duke Clinical Research Institute Duke University School of Medicine Durham, NC United States; ^2^ Lumiata, Inc San Mateo, CA United States; ^3^ Clinical Research Division West Health Institute La Jolla, CA United States; ^4^ University of Texas Health Science Center Houston, TX United States; ^5^ Clinical Research Division Scripps Translation Science Institute La Jolla, CA United States

Nick Peterson, PhD (Lumiata, Inc, San Mateo, CA, United States ) was inadvertently omitted from the list of authors in “A Web-Based Tool for Patient Triage in Emergency Department Settings: Validation Using the Emergency Severity Index” (JMIR Med Inform 2015;3(2):e23). The author Nick Peterson should have been added after Kim Branson in the original published manuscript. This error has been corrected in the online version of the paper on the JMIR Med Inform website on August 11, 2015, together with publishing this correction notice. This was done after submission to PubMed Central and other full-text repositories. This correction notice has been submitted to PubMed, the original paper resubmitted to PubMed Central, and the metadata has been resubmitted to CrossRef with publishing this correction notice.

